# 2-Hydroxypropyl-gamma-cyclodextrin overcomes NPC1 deficiency by enhancing lysosome-ER association and autophagy

**DOI:** 10.1038/s41598-020-65627-4

**Published:** 2020-05-26

**Authors:** Ashutosh Singhal, Evan S. Krystofiak, W. Gray Jerome, Byeongwoon Song

**Affiliations:** 1 Department of Microbiology, Immunology, and Physiology, Meharry Medical College, Nashville, TN 37208 USA; 2 Department of Cell and Developmental Biology, Vanderbilt University, Nashville, TN 37232 USA; 3 Department of Pathology, Microbiology, and Immunology, Vanderbilt University School of Medicine, Nashville, TN 37232 USA

**Keywords:** Cell biology, Diseases

## Abstract

Niemann-Pick type C (NPC) disease is a fatal neurodegenerative disorder caused by mutations in *NPC1* and *NPC2* genes that result in an accumulation of cholesterol in lysosomes. The majority of children with NPC die in adolescence. Currently, no FDA-approved therapies exist for NPC and the mechanisms of NPC disease are not fully understood. Our recent study and the reports from other laboratories showed that 2-hydroxypropyl-γ-cyclodextrin (HPγCD) alleviates cholesterol accumulation in NPC1-deficient cells in spite of its low binding affinity for cholesterol. In this study, we explored the cellular changes that are induced upon HPγCD treatment in NPC1 patient-derived fibroblasts. We show that HPγCD treatment increases lysosome-ER association and enhances autophagic activity. Our study indicates that HPγCD induces an activation of the transcription factor EB (TFEB), a master regulator of lysosomal functions and autophagy. Lysosome-ER association could potentially function as conduits for cholesterol transport from lysosomes to the ER. Accumulating evidence suggests a role for autophagy in rescuing the cholesterol accumulation in NPC and other degenerative diseases. Collectively, our findings suggest that HPγCD restores cellular homeostasis in NPC1-deficient cells via enhancing lysosomal dynamics and functions. Understanding the mechanisms of HPγCD-induced cellular pathways could contribute to effective NPC therapies.

## Introduction

Mutations in NPC1 or NPC2 lead to Niemann-Pick type C (NPC) disease – a fatal lysosomal storage disorder with progressive neurodegeneration^1^. Currently, no FDA-approved therapies exist for the NPC disease with the majority of NPC patients dying before age 20. Clinical symptoms of NPC include hepatic dysfunction, ataxia, spasticity, and dementia^1^. At the cellular level, NPC1- or NPC2-deficient cells accumulate an excessive amount of cholesterol and various sphingolipids in late endosomes and lysosomes, leading to disruptions in protein and lipid trafficking^2,3^. After entering the cell via endocytosis, dietary low-density lipoprotein (LDL)-derived cholesterol is transported into early endosomes, late endosomes, and lysosomes. Cholesterol is then exported from the late endosomes/lysosomes to other cellular compartments, including the endoplasmic reticulum (ER), which senses intracellular sterol content and modulates endogenous cholesterol biosynthesis^4^. The exit of cholesterol from the late endosomes/lysosomes is known to involve the concerted action of NPC1^5^ and NPC2^6^ while the detailed mechanism of cholesterol trafficking remains to be defined. Both NPC1 and NPC2 are present in late endosomes and lysosomes, in which NPC1 resides in the membrane while NPC2 is localized within the lumen of late endosomes/lysosomes^7,8^. Mutations in NPC1 and NPC2 are responsible for ~95% and ~5% of NPC disease, respectively^9^. In addition to lysosomal cholesterol accumulation, NPC1-deficient cells exhibit defects in autophagy^10^, which is a critical cellular process responsible for removing cytoplasmic macromolecules and damaged organelles through the function of lysosomal enzymes. Thus, NPC1 deficiency seems to impair both lysosomal cholesterol trafficking and the autophagy-lysosomal pathway. These findings suggest that there is a link between the autophagy-lysosomal pathway and the NPC disease mechanisms. In support of this notion, recent studies showed that methyl-βCD can normalize cholesterol homeostasis in NPC1-deficient cells by promoting autophagy through AMP-activated protein kinase (AMPK) pathway^11^. The autophagy pathway becomes particularly important under conditions of proteotoxic stress^12^. Deficiency in autophagic function contributes to accumulation of aggregated proteins and are implicated in a range of neurodegenerative disorders such as Parkinson’s disease (PD) and Huntington’s disease (HD)^13,14^. Thus, strategies to enhance autophagy-lysosomal functions could improve cellular homeostasis and contribute to effective therapeutics for NPC disease as well as a number of neurodegenerative disorders.

Lysosomes are dynamic organelles that not only mediate degradation of cellular substrates but also participate in a number of cellular homeostasis processes such as plasma membrane repair, antigen presentation, cell migration, autophagy, and cholesterol homeostasis^15^. The lysosomal responses to different environmental stimuli are regulated by a gene network under the control of a master transcription factor EB (TFEB). TFEB induces the expression of lysosomal genes by binding to the ‘coordinated lysosomal expression and regulation’ (CLEAR) sites in the promoters of TFEB target genes – the CLEAR network^16^. While phosphorylated TFEB is bound by 14-3-3 proteins and retained in the cytoplasm, TFEB translocates to the nucleus upon dephosphorylation and induces expression of the CLEAR gene network^16^, which are involved in lysosomal biogenesis and autophagy. Lysosomes move in both directions between the cell center and the periphery along the microtubule tracks^15,17^. Lysosomal movements within a cell rely on the functions of microtubule motors of the dynein and kinesin families, which are responsible for driving retrograde and anterograde transport of lysosomes, respectively^18,19^. Disruption in these opposing movements results in lysosome clustering at the peripheral or perinuclear areas of the cell, respectively. Previous reports indicated that cholesterol accumulation in NPC1-deficient cells is associated with perinuclear clustering of lysosomes^20–22^. We recently demonstrated that 2-hydroxypropyl-γ-cyclodextrin (HPγCD) treatment promotes a wide distribution of lysosomes across the cytoplasm in NPC1 patient-derived fibroblasts, whereas lysosomes are clustered near the cell center in untreated cells^23^, raising the possibility that HPγCD has the potential to modulate lysosomal dynamics and functions. Lysosome-ER membrane contacts are implicated as an important mechanism for lysosome positioning^24^ and lipid transport^25^. The protein tethers present at lysosome-ER contact sites include oxysterol binding protein related protein (ORP) 1L-vesicle associated membrane protein (VAMP)-associated protein [VAP], steroidogenic acute regulatory protein (StAR) D3 [STARD3]-VAP, and NPC1-ORP5 proteins^25^. A recent study showed a role of NPC1 in tethering ER-endocytic organelle contacts where it interacts with the ER sterol transport protein Gramd1b to regulate cholesterol egress^26^.

HPβCD is known to ameliorate NPC disease phenotype in NPC mouse models^27–30^ and human patients^31,32^. While HPβCD has an ability to bind to cholesterol, the detailed mechanism of action and molecular target of HPβCD are not known. Furthermore, reports of HPβCD-induced ototoxicity in mice and cats^33–35^ have been a major concern in moving HPβCD forward as a therapeutic agent. Recently, HPγCD was shown to reduce cholesterol accumulation in NPC cellular and mouse models^36^. Studies by Davidson *et al*. further demonstrated that HPγCD shows an efficacy in NPC mice equivalent to HPβCD but the ototoxicity and cholesterol-binding capacity of HPγCD are significantly lower than those of HPβCD^37^. My laboratory recently demonstrated that HPγCD and HPβCD alleviate cholesterol accumulation in NPC1 patient-derived fibroblasts^23,38^ and the ability of HPγCD to solubilize cholesterol is significantly lower compared to that of HPβCD^38^. These findings suggest that HPγCD may restore cholesterol homeostasis by inducing broad cellular signaling mechanisms instead of directly extracting cholesterol from the cell membranes. Our proteomics study showed that HPβCD and HPγCD induce a common set of proteins as well as a distinct set of proteins^23^ suggesting that some of the proteins or pathways involved in the control of cellular cholesterol homeostasis might be overlapping between HPβCD and HPγCD. Taken together, HPγCD might be a safer CD that can shed light on NPC disease mechanisms as well as on new therapeutic approaches for the NPC disease.

In this report, we investigated HPγCD-induced changes in NPC1 patient-derived fibroblasts to understand the molecular mechanisms of NPC disease. Here, we show that HPγCD treatment increases lysosome-ER association and enhances the autophagy-lysosomal pathway, resulting in an improvement in cholesterol and cellular homeostasis under conditions of NPC1 deficiency. Of interest, the treatment with HPγCD promoted the activation of TFEB, a master regulator of lysosomal functions. Collectively, these data suggest that HPγCD overcomes NPC1 deficiency via enhancing lysosomal dynamics and functions. Elucidating the mechanisms by which HPγCD restores lysosomal homeostasis and relieves cellular stress will provide new therapeutic approaches for NPC disease and related lysosomal storage disorders.

## Results

### HPγCD treatment enhances cellular homeostasis in NPC1 fibroblasts

**Figure 1:**
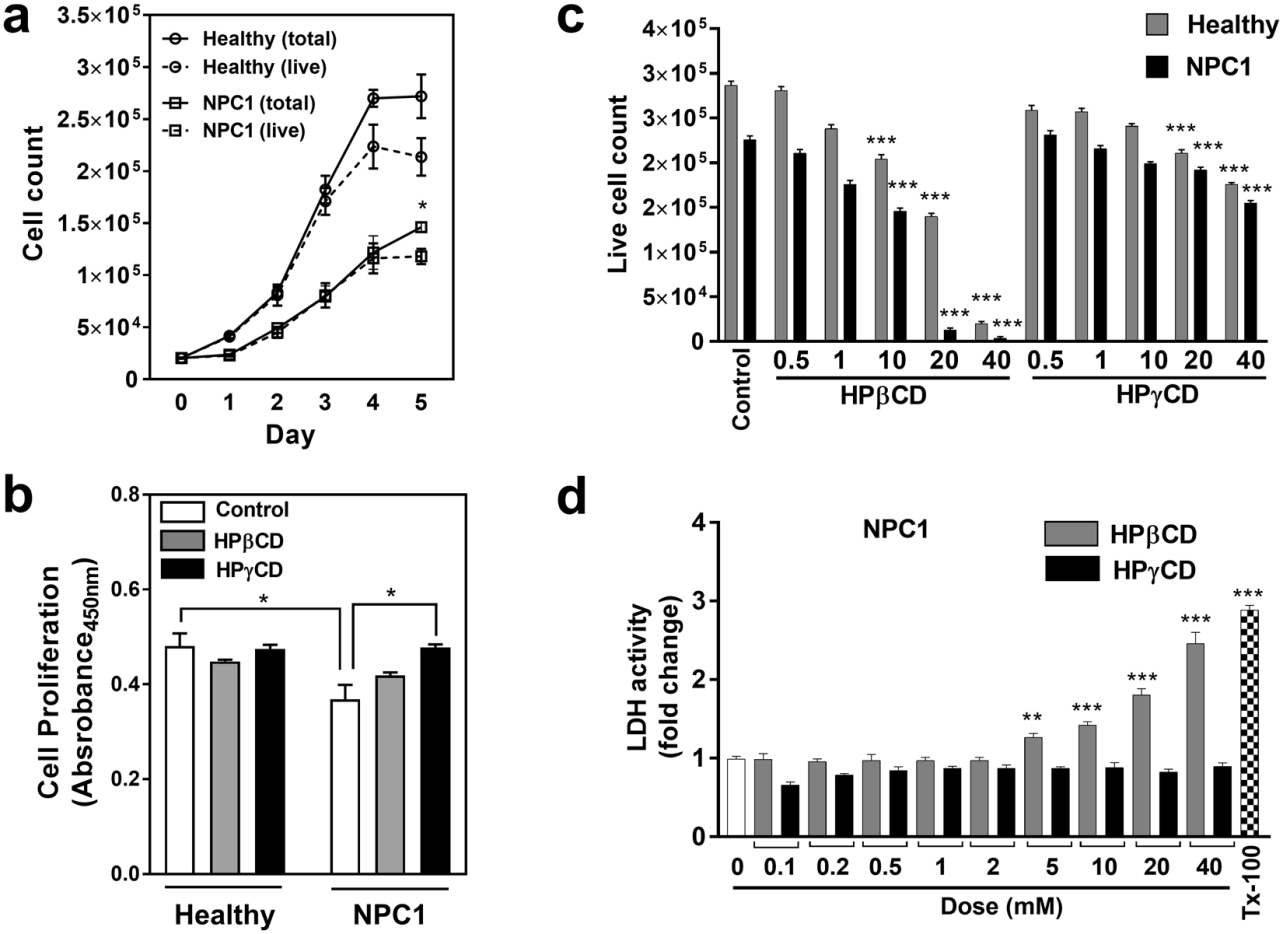
HPγCD enhances cellular homeostasis in NPC1 fibroblasts. Skin fibroblasts from a healthy donor (Healthy) or NPC1 patient (NPC1) were examined for cell proliferation (**a**,**b**) or cell cytotoxicity (**c**,**d**) following treatment without or with HPβCD or HPγCD. Cells were seeded and counted every day for up to 5 days (**a**). Growth kinetics showed that NPC1 cells do not grow as rapidly as healthy cells. To compare proliferation rates, both healthy and NPC1 cells were treated with HPβCD or HPγCD (1 mM, 48 h) and labelled with BrdU for 24 h (**b**). The BrdU assay showed that NPC1 mutant cells proliferate at slower rate as compared to healthy cells. HPγCD treatment improved the cell proliferation rate in NPC1 mutant cells whereas CD treatment had no effect on cell proliferation in healthy cells. Data are mean ± S.E.M. of triplicates and a representative of three independent experiments (*P < 0.05). Cells were treated with 0.5–40 mM of either HPγCD or HPβCD for 48 h. Cytotoxicity was measured by cell counting (**c**) or LDH assay (**d**). The trypan blue positive cells were subtracted from total cell count and referred as live cells. LDH assay was presented as fold change in LDH activity in reference to untreated cells. Triton X-100 (Tx-100) (0.1% v/v) was used as a positive control for cytotoxicity. Data are mean ± S.E.M. of triplicates and a representative of three independent experiments. Symbols indicate the relative level of significance compared with control (**P < 0.01, ***P < 0.001).

We hypothesized that the accumulation of unesterified (free) cholesterol in the lysosomes of NPC1-deficient cells would interfere with lysosomal functions, resulting in an impairment of cellular homeostasis. To test this hypothesis, we cultured primary fibroblasts derived from a NPC1 patient or healthy control and measured cell numbers over time. In support of our hypothesis, the numbers of both live and total cells increased over time in NPC1 fibroblasts at an almost 2-fold slower rate compared with the cells from a healthy control (Fig. 1a). Next, we determined whether NPC1 deficiency leads to a compromise in cell proliferation using the BrdU assay. The proliferation of NPC1-deficient cells was slower compared with the cells from a healthy control and the treatment with HPγCD (1 mM, 48 h) promoted cell proliferation in NPC1 cells whereas cells from a healthy control were not affected by the CD treatment (Fig. 1b). These results suggest that NPC1 deficiency results in an impairment in cellular homeostasis that could be restored by the HPɣCD administration. Next, we determined the dose-dependent effects of HPγCD on fibroblasts derived from a NPC1 patient and healthy control. HPβCD was included for a comparison. The number of live cells was reduced by HPβCD at 10 mM or higher concentration whereas the inhibitory effect of HPγCD on cell growth was only detected at 20 mM or higher concentration (Fig. 1c). The differences in cytotoxic activity between HPβCD and HPγCD were more evident at 20 and 40 mM, in particular for NPC1 mutant cells, suggesting a higher cytotoxic activity of HPβCD compared to HPγCD. The cytotoxic capacity of HPβCD and HPγCD was further evaluated by the lactate dehydrogenase (LDH) assay (Fig. 1d). The release of LDH into the culture medium was higher in cells treated with HPβCD at 5 mM or higher concentration compared to the untreated control, whereas HPɣCD treatment did not influence the release of LDH at the concentrations tested in this study. Finally, we evaluated the morphology of the NPC1 mutant cells and healthy control cells following the treatment with the CDs for 72 h. Our data indicate that HPβCD exerts a cytotoxic effect at much lower concentrations compared with HPɣCD (Fig. S1). Taken together, these results suggest that HPɣCD can rescue the cellular stress of NPC1 deficiency while exhibiting a much less cytotoxic profile compared with HPβCD. We previously tested 12 CD derivatives for their cytotoxic effects on a number of human cell lines and demonstrated that most of the CDs including HPβCD and HPγCD do not exert cytotoxic effect at 1 mM or lower concentration^38^. Therefore, in the subsequent experiments of this study, we treated cells with HPβCD and HPγCD at 1 mM concentration for 48 h or 72 h since both times had mostly similar effect.

### HPγCD alleviates intracellular cholesterol accumulation in NPC1 fibroblasts

**Figure 2:**
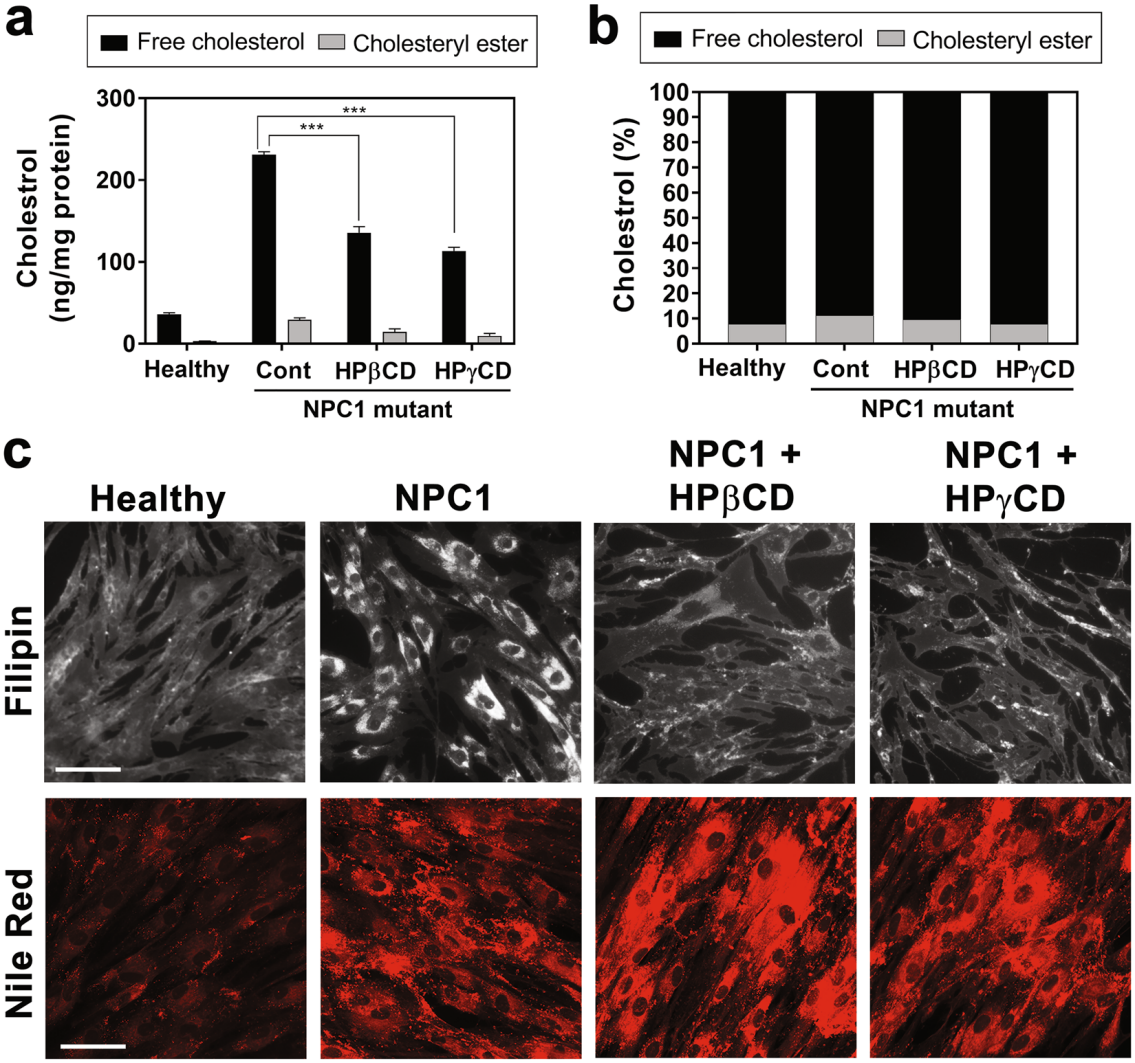
HPγCD alleviates cholesterol accumulation in NPC1 fibroblasts. Skin fibroblasts from a healthy donor (Healthy) or NPC1 patient (NPC1) were treated for 72 h with HPβCD (1 mM) or HPγCD (1 mM) and then examined for cholesterol and neutral lipid content. The levels of cholesterol in cell lysates were measured by cholesterol assay (**a**,**b**) in a reaction mixture with (for total cholesterol content) or without (for free cholesterol content) cholesterol esterase enzyme. The intracellular accumulation of free cholesterol and neutral lipid were evaluated by staining with Filipin and Nile Red, respectively (**c**). Data are mean ± S.E.M. of triplicates and a representative of three independent experiments. Symbols indicate the relative level of significance compared with the control (***P < 0.001). Scale bar = 50 µm.

The accumulation of unesterified cholesterol in late endosomes and lysosomes is a prominent feature of NPC1 deficiency, reflecting the defects in lysosomal cholesterol trafficking. We previously demonstrated that HPγCD treatment rescues cholesterol accumulation in NPC1-deficient cells in spite of very low cholesterol-binding ability^23,38^. To evaluate the changes in the cellular cholesterol content upon HPγCD treatment, we quantified the cellular cholesterol levels in NPC1 patient cells or healthy control cells cultured in the absence or presence of HPβCD (1 mM, 72 h) or HPγCD (1 mM, 72 h) using the Total Cholesterol Assay Kit (Cell Biolabs, San Diego, CA). This assay allows to detect total cholesterol in the presence of cholesterol esterase or only free cholesterol in the absence of the esterase enzyme; the levels of cholesteryl ester are determined by subtracting the levels of free cholesterol from the levels of total cholesterol. As expected, the levels of free cholesterol in NPC1-deficient cells were much higher compared to healthy control cells and the treatment with HPβCD or HPγCD significantly reduced the levels of free cholesterol in NPC1-deficient cells (Fig. 2a). The cellular content of cholesteryl ester was less than 10% under all conditions tested (Fig. 2b). By using fluorescence microscopy, we examined intracellular accumulation of free cholesterol and neutral lipids. The treatments with HPβCD (1 mM, 72 h) and HPγCD (1 mM, 72 h) reduced the accumulation of free cholesterol as probed with filipin staining (Fig. 2c, top), with a concomitant increase in neutral lipid-enriched membranes as probed with Nile red staining (Fig. 2c, bottom). These results suggest that HPγCD rescues the lysosomal cholesterol accumulation in NPC1-deficient cells as efficiently as HPβCD does.

With the cholesterol assay system used in our study, the cholesteryl ester content was not increased upon the CD treatment (Fig. 2a,b). These unexpected results might be due to the limitation of the cholesterol assay system, which relies on the activity of the esterase enzyme added during the assay for measuring the levels of cholesteryl ester. Additional experiments are necessary to determine whether the increase in the nonselective neutral lipids stained with Nile Red upon the CD treatment has a role in the rescue of NPC1 deficiency by CD treatment.

### HPγCD rescues the structural abnormalities of lysosomes and promotes lysosome-ER association in NPC1 fibroblasts

**Figure 3:**
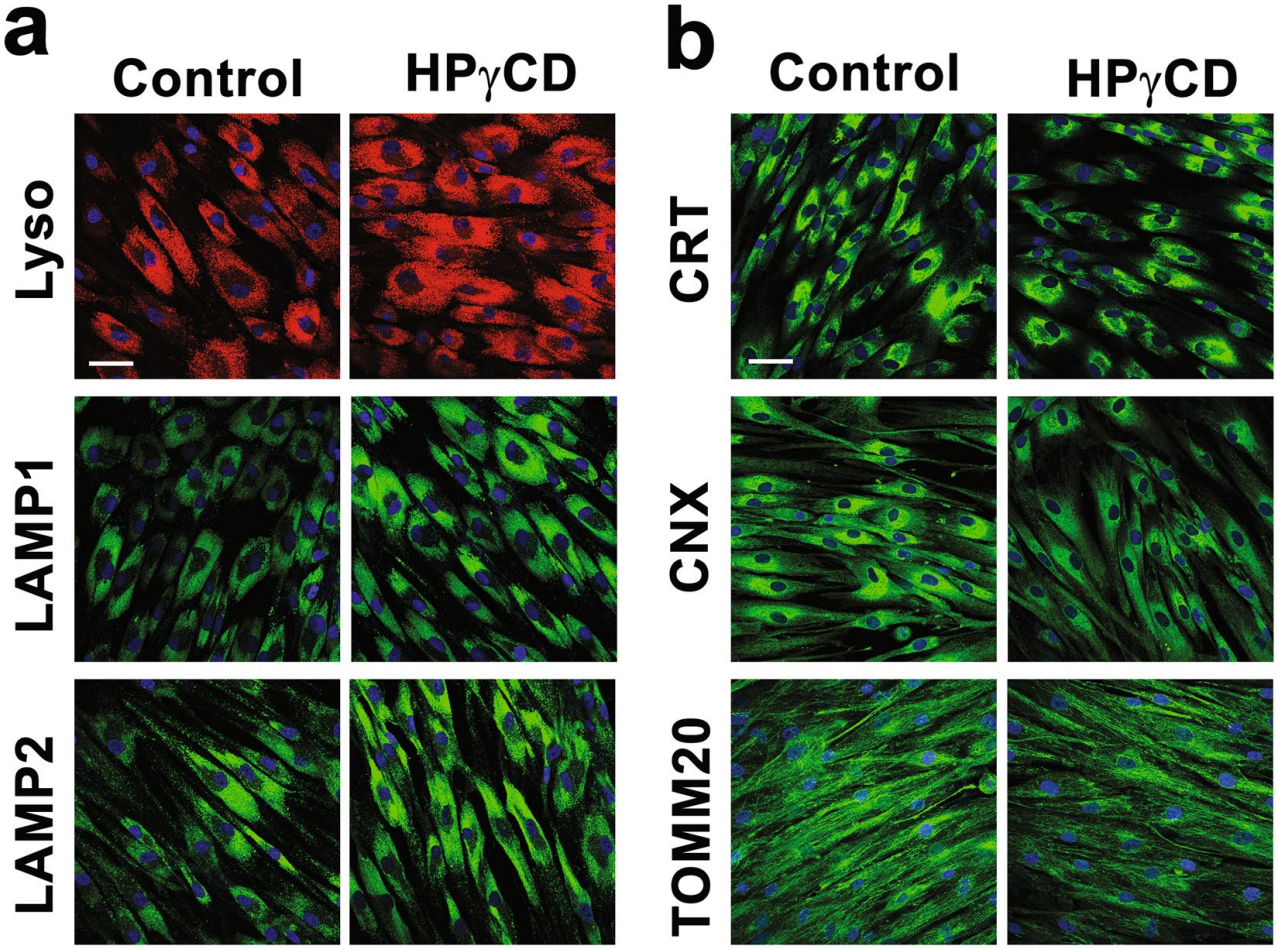
Effect of HPγCD treatment on lysosomes, ER, and mitochondria in NPC1 fibroblasts. The characteristics of lysosomes, ER, and mitochondria of NPC1 mutant cells were examined by confocal microscopy following the treatment with HPγCD (1 mM, 72 h). The cellular distribution of lysosomes was analyzed by live-cell labeling with LysoTracker Red or staining fixed cells (4% paraformaldehyde) with LAMP1 or LAMP2 antibodies (**a**). The ER was analyzed by staining with calreticulin (CRT) or calnexin (CNX) antibodies and mitochondria were detected by staining with translocase of outer mitochondrial membrane 20 (TOMM20) antibody (**b**). Data are representative of three independent experiments. Scale bar = 50 µm.

**Figure 4:**
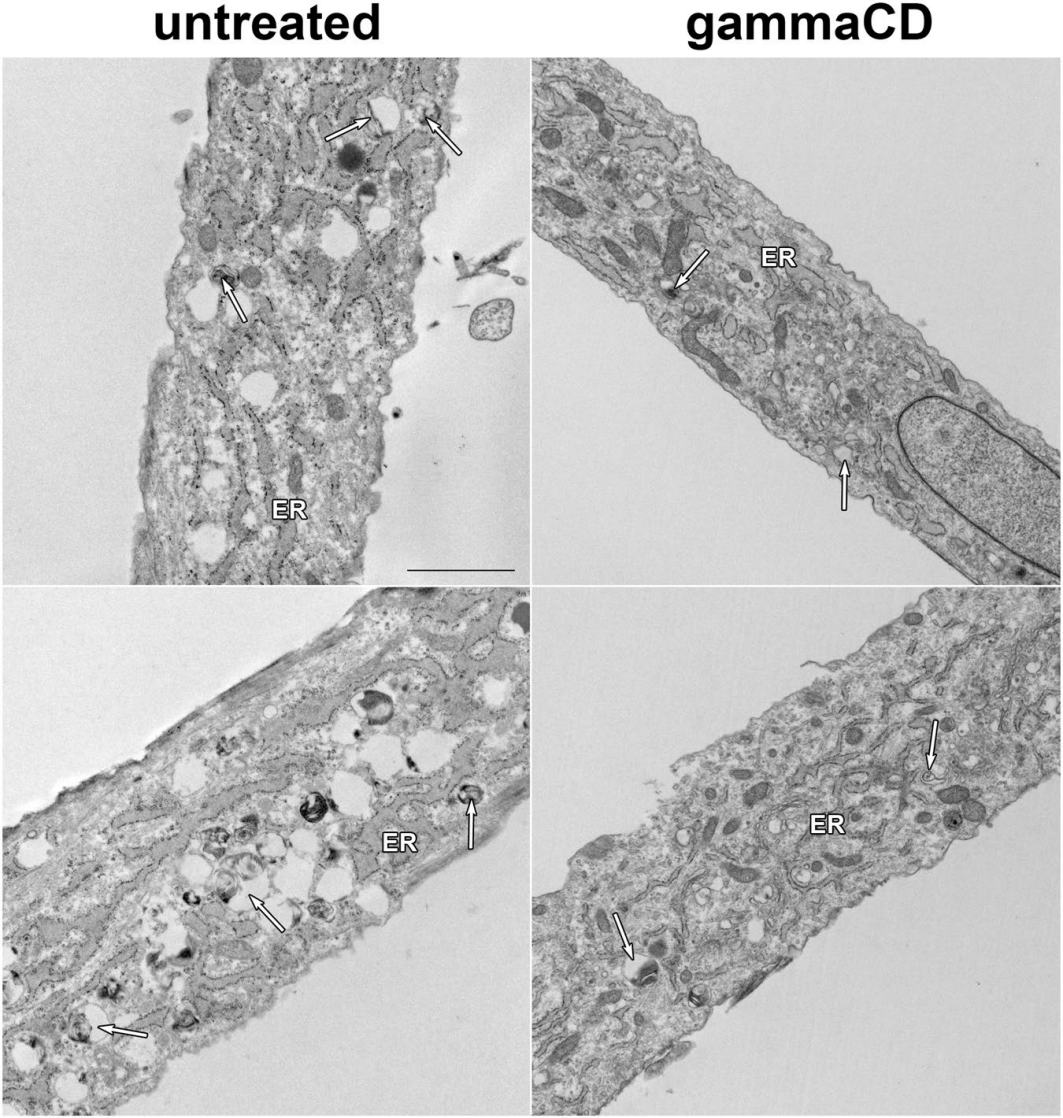
HPγCD rescues the structural abnormalities of lysosomes and ER in NPC1 fibroblasts. Transmission electron microscopy of untreated NPC1 fibroblasts (left column) and NPC1 fibroblasts treated with HPγCD (1 mM, 72 h) (right column). Untreated cells showed large lysosomes with many of the lysosomes being lipid-engorged (arrows). The endoplasmic reticulum (ER) of untreated cells were swollen with filled lumens. In contrast, following treatment with HPγCD, the cells had more normal appearing endoplasmic reticulum and the lysosomes were less lipid-engorged. All images magnified 9,000×. Black bar = 2 micrometers.

We hypothesized that a massive accumulation of unesterified cholesterol in the lysosomes of NPC1-deficient cells could result in an alteration in the structures and/or intracellular distribution of the lysosomes and other organelles. First, we determined the subcellular expression of lysosomes, ER, and mitochondria in NPC1 fibroblasts treated without or with HPγCD (1 mM, 72 h) by live-cell imaging and confocal microscopy (Fig. 3). Upon HPγCD treatment, lysosomes were distributed more widely across the cytoplasm and the signals of lysosomal markers were more intense compared to untreated cells (Fig. 3a); these results are consistent with our previous report showing enhancement of LAMP1 expression by HPγCD treatment^23^. On the other hand, HPγCD treatment did not influence the signals of the ER and mitochondria markers compared to untreated cells (Fig. 3b). Next, we conducted transmission electron microscopy to probe the structural changes in lysosomes and ER in NPC1 fibroblasts following the treatment with HPγCD (Fig. 4). Untreated NPC1 fibroblasts showed large lysosomes with many of the lysosomes being lipid-engorged. The ER of untreated NPC1 cells were generally swollen with filled lumens. Importantly, following the treatment with HPγCD, the NPC1 cells had more normal appearing lysosomes and ER, with the lysosomes being less lipid-engorged. Collectively, these data indicate that NPC1 deficiency leads to the structural abnormalities of lysosomes and ER and suggest that HPγCD can potentially rescue the structural and functional defects of lysosomes and ER under conditions of NPC1 deficiency.

**Figure 5:**
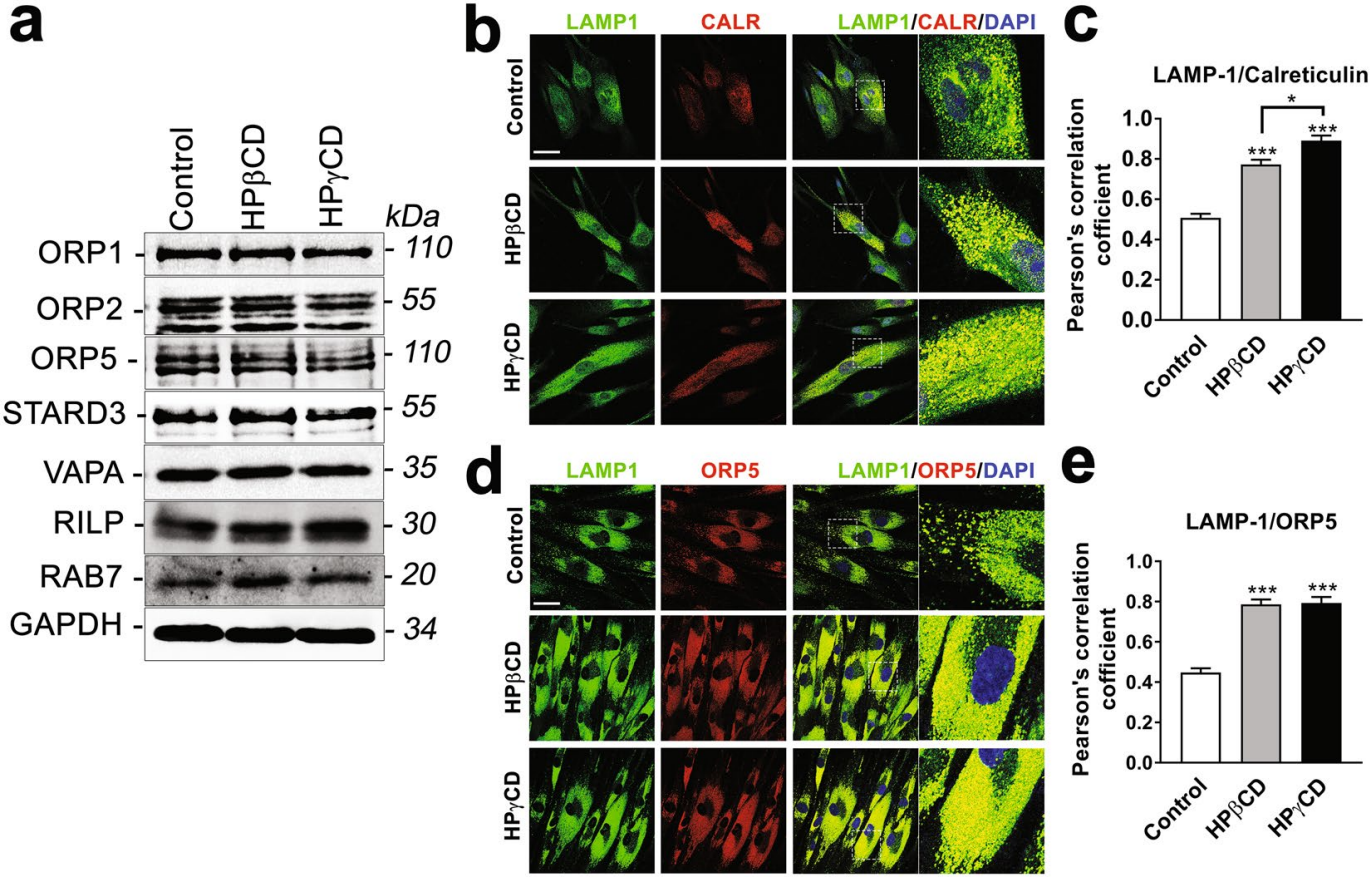
HPγCD enhances lysosome-ER association in NPC1 fibroblasts. NPC1 mutant cells were treated with HPγCD or HPβCD (1 mM, 72 h). Cells were then analyzed for the levels of lysosome and ER contact proteins by immunoblotting (**a**) and for lysosome-ER association by confocal microscopy (**b–e**). The blots are from different parts of the same gel and delineated with dividing lines. Confocal microscopy was used to visualize the lysosome and ER by immunostaining cells for the lysosomal membrane protein (LAMP1, green) and the ER protein (Calreticulin or ORP5, red). Microscopic images showed co-localization of LAMP1 and CALR (**b**) or ORP5 (**d**) as a yellow color (merge). Nuclei were stained using DAPI (blue). The co-localization, measured by Pearson’s correlation coefficient, was significantly higher in the CD-treated cells compared to untreated control cells (**c,e**). Data are mean ± S.E.M. of triplicates and a representative of three independent experiments. Symbols indicate the relative level of significance compared with the control (*P < 0.05, ***P < 0.001). Scale bar = 50 µm.

Despite its low cholesterol content, the ER plays a crucial function in the intracellular distribution of cellular cholesterol and makes extensive contacts with various cellular compartments such as the plasma membrane, endosomes/lysosomes, the Golgi complex, and lipid droplets^39^. The membrane contact sites between the ER and endosomes/lysosomes are particularly abundant, suggesting important physiological roles including lipid transfer and calcium signaling^24^. Recent studies demonstrated several protein tethers or interactions at the lysosome-ER contact sites that could potentially mediate lipid trafficking between the two organelles; these protein-protein interactions include ORP1L-VAP, STARD3-VAP, NPC1-ORP5, and NPC1-Gramd1b protein complexes^26,40^. Lysosome-ER membrane contacts could function as conduits for a direct, non-vesicular transfer of lysosomal cholesterol to the ER. We determined whether HPγCD treatment influences lysosome-ER association in NPC1-deficient cells. Using immunoblotting assay, we first confirmed that the proteins that are potentially involved in lysosome-ER tethering are expressed at equivalent levels in NPC1-deficient cells treated without and with HPβCD or HPγCD (Fig. 5a). Using confocal microscopy, we demonstrated that the treatments with HPβCD and HPγCD promote the association of lysosomes with the ER, as shown by the co-localization of the lysosomal protein LAMP1 with the ER protein calreticulin (Fig. 5b,c) or ORP5 (Fig. 5d,e). These results suggest that the treatments with HPβCD and HPγCD increase lysosome-ER association, potentially facilitating a direct, non-vesicular trafficking of cholesterol from the lysosomes to the ER. HPγCD-induced lysosome-ER association could be an important mechanism for lysosomal cholesterol exit, independent of NPC1 function. The association of lysosomes with mitochondria (Fig. S2) or with peroxisomes (Fig. S3) was not affected by the treatments with HPβCD and HPγCD.

### HPγCD promotes autophagic function in NPC1 fibroblasts

**Figure 6:**
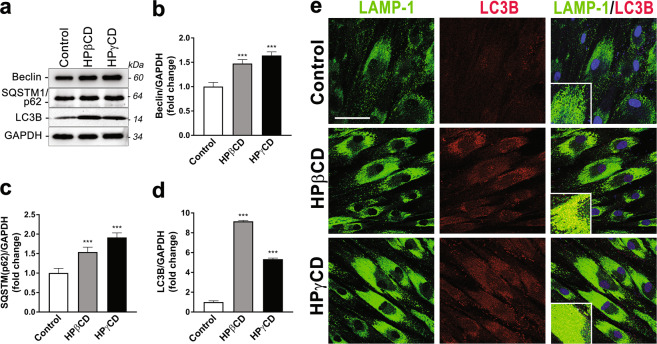
HPγCD promotes autophagy in NPC1 fibroblasts. NPC1 mutant cells treated with HPγCD or HPβCD (1 mM, 72 h) were analyzed for expression of autophagy marker proteins by Western blot and confocal microscopy. Cells were lysed and immunoblotted for Beclin-1, SQSTM1/p62, or LC3B (**a**). The blots are from different parts of the same gel and delineated with dividing lines. Western blot was analyzed using GAPDH as a loading control and the fold changes in protein expression levels were calculated using densitometry. Protein levels of Beclin-1 (**b**), SQSTM1/p62 (**c**), and LC3B (**d**) were significantly increased by HPγCD or HPβCD treatment. Co-localization of autophagosome and lysosome markers was analyzed by confocal microscopy (**e**). Association of LAMP-1 (lysosome marker, green) with LC3B (autophagosome marker, red) was enhanced in HPγCD or HPβCD treated cells. Data are mean ± S.E.M. of triplicates and a representative of three independent experiments. Symbols indicate the relative level of significance compared with the control (***P < 0.001). Scale bar = 50 µm.

We determined the impact of HPγCD treatment on autophagic activity, which is defective under conditions of NPC1 deficiency. To this end, we examined three proteins involved in different steps of the autophagic pathway using immunoblotting analysis: these include microtubule-associated proteins 1 A/1B light chain 3 (LC3), essential for the formation of autophagic vesicles; SQSTM1/p62, essential for cargo recognition; and Beclin-1, required for the formation of autophagosomes^41^. We observed an increase in the levels of Beclin 1, SQSTM1/p62, and LC3 proteins in NPC1 fibroblasts upon treatment with HPγCD or HPβCD compared to untreated control cells (Fig. 6a). The effects of the CDs on the expression of LC3 (Fig. 6d) were more pronounced compared to their effects on the expression of Beclin 1 (Fig. 6b) or SQSTM1/p62 (Fig. 6c). Confocal microscopy analysis confirmed the induction of LC3B expression upon treatment with the CDs (Fig. 6e). LC3 is a protein found on autophagosomal membranes^42^ and is widely used as a marker of autophagy activation^43^. Thus, our findings suggest that the treatments with HPγCD and HPβCD could promote the autophagy pathway in NPC1-deficient cells, while we cannot exclude the possibility that the observed increase in autophagosomes could be linked to a secondary reduction of autophagy flux. We anticipate that HPγCD activation of the autophagy pathway will enhance the cellular clearance system, alleviating the cellular stress of NPC1 deficiency.

**Figure 7:**
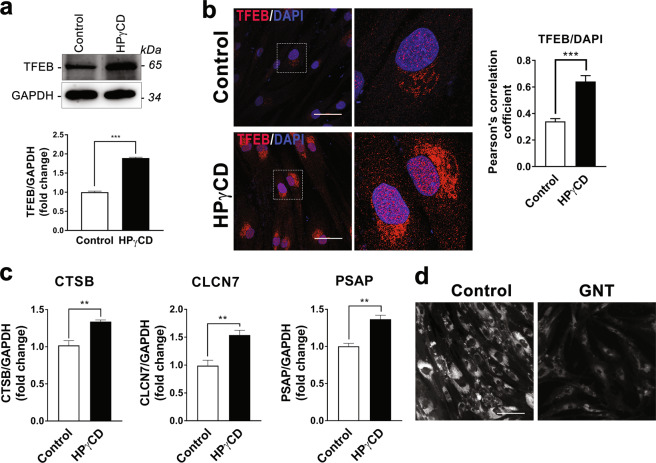
HPγCD promotes TFEB activation in NPC1 fibroblasts. TFEB expression, activation and the induction of TFEB target (the CLEAR network) were evaluated in NPC1 fibroblasts following the treatment without or with HPγCD (1 mM, 48 h). Cells were lysed and immunoblotted for TFEB (**a**). The blots are from different parts of the same gel and delineated with dividing lines. The HPγCD-treated cells showed significantly higher TFEB protein levels as calculated by densitometry analysis. TFEB activation was evaluated by nuclear localization of TFEB as analyzed by confocal microscopy (**b**). Microscopic images showed nuclear localization of TFEB as a purple color (merge) resulted from co-localization of TFEB (red) and DAPI (blue). The co-localization was measured by Pearson’s coefficient. Real-time PCR was used to analyze the relative mRNA expression levels of TFEB target genes in NPC1 mutant cells following the treatment without or with HPγCD (1 mM, 48 h). The expression levels of the members of CLEAR gene network (CTSB, CLCN7 and PSAP) were calculated by considering GAPDH as a reference gene and data was presented as fold changes in expression as compared to untreated cells (**c**). The effect of genistein (GNT; 25 μM, 48 h) on intracellular accumulation of free cholesterol in NPC1 mutant cells was evaluated by staining with Filipin (**d**). Data are mean ± S.E.M. of triplicates and a representative of three independent experiments. Symbols indicate the relative level of significance compared with the control (**P < 0.01, ***P < 0.001). Scale bar = 50 µm.

To understand the molecular mechanisms by which HPγCD enhances the autophagy-lysosomal pathway, we probed the expression and activity of the transcription factor TFEB, a master regulator of lysosomal biogenesis and autophagy^16^. Treatment of NPC1 patient-derived fibroblasts with HPγCD enhanced the expression of TFEB (Fig. 7a). We next examined the nuclear translocation of TFEB as a measure of TFEB activation. Upon treatment of NPC1 cells with HPγCD, we observed an increase in the levels of TFEB localized in the nuclei compared to untreated control cells (Fig. 7b). The nuclear translocation of TFEB suggested an induction of its target genes-the CLEAR network. To test this possibility, we measured the expression of representative genes of the CLEAR network. NPC1-deficient cells were treated with HPγCD and the mRNA expression levels of TFEB target genes were monitored by quantitative RT-PCR. We observed an enhancement in the expression of TFEB target genes, namely, *CTSB* [cathepsin B; 1.34-fold], *CLCN7* [chloride channel 7; 1.54-fold], and *PSAP* [prosaposin; 1.37-fold] (Fig. 7c) upon HPγCD treatment compared with their expression in untreated NPC1 cells. Interestingly, a recent study showed that HPβCD treatment enhances autophagy through the activation of TFEB in the model of another lysosomal storage disorder, neuronal ceroid lipofuscinosis^44^. Finally, we tested the functional significance of TFEB activation in the rescue of the NPC phenotype by using the phytoestrogen genistein, which is known to induce TFEB activation and autophagy^45,46^. Our data indicate that the treatment with genistein (25 μM, 48 h) significantly alleviates the intracellular accumulation of free cholesterol in NPC1 fibroblasts (Fig. 7d) without exerting any adverse effect on cell viability (Fig. S4). Taken together, these results suggest that TFEB could play an important role in HPγCD-mediated enhancement of the autophagy-lysosomal pathway and cellular homeostasis under conditions of NPC1 deficiency. We anticipate that TFEB activation and the subsequent lysosomal biogenesis/autophagy induction could play a crucial function in rescuing the cholesterol accumulation and cellular stress in NPC1-deficient cells.

## Discussion

**Figure 8:**
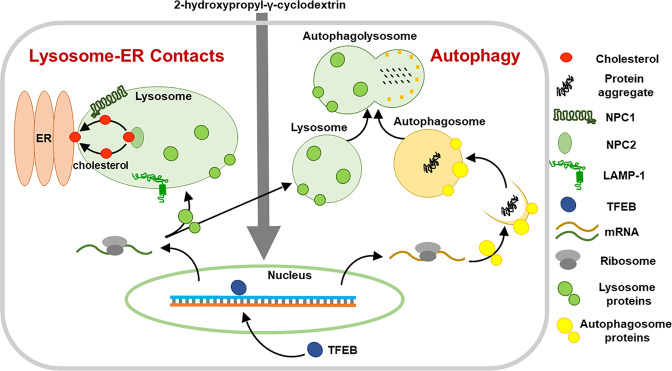
A proposed model of cellular response to HPγCD treatment. Administration of HPγCD results in activation of TFEB. Upon translocation from the cytoplasm to the nucleus, TFEB induces the expression of genes involved in lysosomal biogenesis and autophagy. As a result, HPγCD treatment results in an enhancement of lysosomal functions, lysosome-ER association, and autophagic activity, which in turn results in the rescue of the cellular stress under conditions of NPC1 deficiency.

NPC disease is caused by mutations in the lysosomal proteins NPC1 or NPC2 and the inflicted individuals suffer from a fatal progressive neurodegeneration^1^. Despite intense studies during the past years, the molecular details of NPC disease are still elusive and effective therapies for NPC are not available at present. In this study, we provide for the first time evidence that links HPγCD induction of lysosomal functions to the rescue of cellular homeostasis under conditions of NPC1 deficiency. Our data indicate that HPγCD alleviates lysosomal cholesterol accumulation and enhances autophagic activity in NPC1-deficient cells. Interestingly, HPγCD promoted lysosome-ER association. Further, our data indicate that HPγCD promotes the activation of TFEB, a master regulator of lysosomal biogenesis and autophagy^16^. Here, we propose a model wherein HPγCD restores cholesterol and cellular homeostasis under conditions of NPC1 deficiency by enhancing lysosomal dynamics and functions (Fig. 8). We provide two potential mechanisms for HPγCD to restore cellular homeostasis in NPC1-deficient cells. First, HPγCD induction of the lysosome-ER association can mediate cholesterol transport from lysosomes to the ER independent of NPC1 function, resulting in lysosomal homeostasis in NPC1-deficient cells. Second, HPγCD enhancement of the autophagy pathway can alleviate the accumulation of toxic protein aggregates in cells, rescuing the cellular stress of NPC1 deficiency. As the completion of autophagy requires the fusion of the autophagosome with the lysosome, we anticipate a crosstalk between autophagy and lysosomal function to maintain cellular homeostasis. As expected, HPβCD rescued the lysosomal cholesterol accumulation in NPC1-deficient cells as efficiently as HPγCD. It is likely that HPβCD executes the NPC1-rescuing functions by extracting cholesterol from cell membranes as well as regulating cellular signaling mechanisms. We previously demonstrated that HPβCD and HPγCD induce the expression of a common set of proteins as well as a distinct set of proteins^23^. Therefore, we anticipate that HPβCD and HPγCD could modulate cellular functions via regulating shared pathways as well as distinct pathways.

Our data show that lysosome-ER association is enhanced in NPC1 cells upon HPγCD treatment. Lysosomes are highly dynamic structures, which change their localization within a cell and their network connectivity with other organelles^13^. Our previous study demonstrated that upon HPγCD treatment, NPC1 cells show a wide distribution of lysosomes across the cytoplasm whereas lysosomes are clustered near the cell center in untreated NPC1 cells^23^. These findings suggest that modulation of lysosomal dynamics may be one of the key mechanisms by which HPγCD restores cellular homeostasis under conditions of NPC1 deficiency. In this study, we show that HPγCD promotes lysosome-ER association in NPC1-deficient cells as shown by co-localization of the lysosomal protein LAMP-1 with the ER proteins calreticulin or ORP5. Accumulating evidence suggests that lysosome-ER association plays a critical role for direct cholesterol transport from lysosomes to the ER^47–49^. A recent study showed that NPC1 regulates ER contacts with late endocytic organelles where it interacts with the ER-localized sterol transport protein Gramd1b and that expansion of the ER-lysosome membrane contact sites is sufficient to rescue the lysosomal cholesterol accumulation in the absence of NPC1^26^. We anticipate that the lysosome-ER association induced upon HPγCD treatment could mediate the transport of lysosomal cholesterol to the ER in the absence of NPC1 function. Cholesterol transport from lysosomes to the ER can be mediated by tethering complexes involving ORP1L or STARD3 at the lysosome and VAP, ORP5, or Gramd1b at the ER. Alternatively, cholesterol transport may occur along a concentration gradient across the contact sites without the need for a sterol transporter. Further experiments are necessary to address these important questions.

Importantly, our findings indicate that HPγCD treatment promotes autophagic function and TFEB activation in NPC1-deficient cells. While NPC1-deficient cells are known to be defective in autophagy^10^, it is not clear how NPC1 deficiency is linked to the disruption of the autophagy pathway. Accumulating evidence suggests a correlation between cholesterol accumulation and autophagy dysfunction in NPC and other degenerative diseases, which are rescued by βCDs^11,50,51^. HPβCD was shown to restore autophagy via rescuing cholesterol accumulation in the models of Alzheimer disease^50^ and Bietti’s crystalline dystrophy^51^, whereas methyl-β-CD (MβCD) was able to rescue impaired autophagy in NPC1-deficient cells via activation of AMPK^11^. We anticipate that autophagy dysfunction in NPC1-deficient cells will promote protein misfolding and aggregation, leading to an increase in cellular stress, in addition to its detrimental effect on cholesterol homeostasis. To this end, our data indicate that NPC1-deficient cells display a slower rate of cell proliferation compared to healthy control cells. Interestingly, the treatment with HPγCD increased the rate of cell proliferation in NPC1-deficient cells. Our data further indicate that HPγCD promotes the expression of the key proteins of the autophagy pathway including Beclin, SQSTM1/p62, and LC3B. These findings suggest that HPγCD has the potential to enhance autophagic functions and cellular clearance, thus improving cellular homeostasis under conditions of NPC1 deficiency. Importantly, our data show that HPγCD promotes the activation of TFEB, a master regulator of lysosomal functions and autophagy^16^. Collectively, our findings suggest that TFEB could play a crucial function in mediating HPγCD-induced rescue of autophagy-lysosomal functions and cellular homeostasis in NPC1-deficient cells. The mechanisms by which HPγCD activates TFEB remains to be defined. HPγCD might be able to enter cells by endocytosis, as demonstrated for a number of βCD derivatives^52–56^. It is also possible that HPγCD may induce cellular signaling upon binding to an unknown receptor on the cell surface. Further work is necessary to clarify these issues. Defects in multiple features of the lysosomal and autophagic network have been implicated in various neurodegenerative and lysosomal storage disorders, raising the possibility that TFEB could be a promising target to restore lysosomal functions under pathological scenario. *In vivo* studies with heterologous expression of TFEB have shown an improvement in clearance and amelioration of pathological conditions in rodent models of neurodegenerative disorders that include Alzheimer’s disease^57,58^, tauopathy^59^, Parkinson’s disease^60^, and Huntington’s disease^61^. Thus, HPγCD induction of autophagy-lysosomal functions could be exploited for therapeutic approaches for NPC disease as well as several other neurodegenerative disorders.

The hallmark of NPC1 deficiency at the cellular level is the accumulation of unesterified free cholesterol in lysosomes. How defects in lysosomal cholesterol trafficking are linked to neurodegeneration in the NPC disease remains to be determined. We speculate that the accumulation of unesterified cholesterol in the lysosomes of NPC1 cells will compromise lysosomal structures and/or functions, resulting in an impairment of cellular homeostasis. In support of this hypothesis, our transmission electron microscopy study demonstrated the abnormal structures of lysosomes in NPC1 patient-derived fibroblasts, with many of the lysosomes being lipid-engorged. Upon HPγCD treatment, the lysosomes became normal-looking with most of the lysosomes less lipid-engorged, suggesting a restoration of lysosomal structures/functions. Interestingly, NPC1-deficient cells also showed swollen ER with filled lumen, indicative of ER stress. Upon treatment with HPγCD, the swollen ER was converted into normal appearing ER. Thus, our data present for the first time evidence that the accumulation of unesterified cholesterol in lysosomes induces the structural alterations of the ER as well as the lysosomes, which are rescued upon HPγCD treatment. These findings suggest that lysosomal cholesterol accumulation may result in an ER stress under conditions of NPC1 deficiency. The functional significance of the abnormal ER structures and/or the ER stress in the mechanisms of NPC disease warrants further study.

The rescue of NPC1 phenotypes by HPγCD is an important finding, considering the known ototoxicity of HPβCD^33–35^. Our previous study showed that the ability of HPγCD to solubilize cholesterol is extremely low, whereas HPβCD efficiently solubilize cholesterol^38^. The work by Soga *et al*. has demonstrated that HPγCD could reduce the cholesterol accumulation and restore the functional and molecular abnormalities in NPC1-deficient cells, more effectively than HPβCD^36^. A recent study by Davidson *et al*. further showed that the mechanism of NPC correction does not strictly correlate with the cholesterol complexation ability of the CDs and HPγCD exhibits an efficacy in NPC model mice with reduced ototoxicity compared to HPβCD^37^. Therefore, the use of HPγCD for enhancing both autophagy-lysosomal functions and cholesterol homeostasis in NPC disease has the unambiguous advantage over applying HPβCD because the potential for HPγCD to evoke ototoxicity and membrane damage is significantly lower compared with HPβCD. In summary, our data show that HPγCD enhances lysosome-ER association, autophagic activity, and lysosomal homeostasis via promoting the activation of the master regulator TFEB, thus restoring cholesterol and cellular homeostasis in NPC1-deficient cells. Since the conclusion on HPγCD-mediated rescue is based only on single cell line derived from one NPC patient and one healthy control, additional experiments using more cell lines should be performed to support the therapeutic interest of HPγCD. Further work is required to uncover the molecular mechanisms that are involved in rescuing the cellular stress under conditions of NPC1 deficiency. We anticipate that the cellular pathways and/or proteins revealed upon HPγCD administration will provide important clues for understanding the NPC disease mechanisms and could serve as new drug targets for effective NPC therapy.

## Materials and Methods

### Reagents

Cell culture media and reagents were purchased from Thermo Fisher Scientific (Waltham, MA). These include Dulbecco’s modified Eagle’s medium (DMEM), MEM non-essential amino acids, fetal bovine serum (FBS), penicillin, and streptomycin. A CellTiter 96 Aqueous One Solution Cell Proliferation Assay System was purchased from Promega (Madison, WI). An LDH Cytotoxicity Assay Kit was obtained from Thermo Fisher Scientific. BrdU Cell Proliferation Assay Kit was purchased from Cell Signaling Technology (Danvers, MA). Filipin III was obtained from Sigma-Aldrich (St. Louis, MO). Nile red and Image-iT Lysosomal and Nuclear Labeling Kit were obtained from Thermo Fisher Scientific. Total cholesterol assay kit (Cell Biolabs) was purchased from VWR (Radnor, PA). Genistein, HPβCD, and HPγCD were obtained from Sigma-Aldrich. Primary antibodies: antibodies for LAMP1 (15665 S, 9091 S), GAPDH (5174 S), Beclin-1 (3495 S), SQTM (88588 S), and LC3B (3868 S) are from Cell Signaling Technology (Danvers, MA); antibodies for calnexin (PIPA534754), ORP2 (PA521891), ORP5 (PA518221), STARD3 (PA1562), VAPA (PA552660), Rab7 (PA5-52369), and RILP (PA534357) are from Thermo Fischer Scientific; antibodies for ORP1L (ab131165), calreticulin (ab92516), and TOMM20 (ab78547) are from Abcam (Cambridge, MA); antibodies for LAMP2 (sc-18822) and TFEB (sc-166736) are from Santa Cruz (Dallas, TX). Secondary antibodies: Horseradish peroxidase (HRP)-conjugated anti-mouse (HAF018) and anti-rabbit (HAF008) are from R&D systems (Minneapolis, MN); CF488A-conjugated goat anti-mouse (20018) and anti-rabbit (20012) and CF594-conjugted goat anti-mouse (20110) and anti-rabbit (20153) are from Biotium (Fremont, CA). TaqMan Fast Advanced Master Mix and TaqMan gene expression probes (GAPDH: Hs03929097; CTSB: Hs00947439; CLCN7: Hs01126462; PSAP: Hs01551096) were purchased from Thermo Fisher Scientific.

### Cell lines and cell culture

Untransformed skin fibroblasts from a patient with NPC1 mutation (GM03123) and fibroblasts from a healthy control (GM05659) were purchased from Coriell Institute (Camden, NJ). The donor subject (9 year-old female) of GM03123 cells is a compound heterozygote; one allele carries a missense mutation resulting in a substitution of a serine for a proline at codon 237 (P237S) and the second allele carries a missense mutation resulting in a substitution of a threonine for an isoleucine at codon 1061 (I1061T). The donor subject of GM05659 is an apparently healthy 1-year old male. Cells were maintained in DMEM (with high glucose, L-glutamine, and sodium pyruvate) containing non-essential amino acids, 10% FBS, 100 U/ml of penicillin, and 100 μg/ml of streptomycin at 37 °C in a 5% CO_2_ humidified incubator.

### Cell counting, cell proliferation, and cytotoxicity

Cell were trypsinized and mixed with equal volume of FBS and twice volume of Trypan Blue Dye (0.4%) and counted by TC20 Automated Cell Counter (Bio-Rad) using disposable counting slides (Bio-Rad). Total and viable cell counts were determined. Cell proliferation was measured using BrdU Cell Proliferation Assay Kit as per the manufacturer’s instructions (Cell Signaling Technology). Cytotoxicity in cells was measured by lactate dehydrogenase assay using the LDH Cytotoxicity Assay Kit (Thermo Fisher Scientific).

### Immunoblotting

The protein levels in cells were determined by immunoblotting as previously mentioned^23^. Briefly, cells were washed with ice cold PBS and lysed in MPER lysis buffer (Thermo Fischer Scientific) containing protease inhibitors cocktail (Thermo Fischer Scientific) for 15 min on ice. The cell debris was removed by centrifuge at 10000 × g for 15 min at 4 °C. The protein levels in clear cell lysate were measured by Bradford assay (VWR Life Science). Total protein (40 μg) was resolved by SDS-PAGE and transferred to nitrocellulose membrane (Bio-Rad). The membrane was blocked in 5% skim milk for 45 min at room temperature followed by probing with 1:1000 diluted primary antibodies (1:250 for ORP5) overnight at 4 °C. After three washes with TBST (50 mM Tris-Cl, 150 mM NaCl, and 0.1% Tween 20; pH7.6), membrane was incubated with respective HRP-conjugated secondary antibodies (1:1000) for 2 h at room temperature and washed three times. The luminescent signal was developed either using Super Signal West Femto Maximum Sensitivity or Dura Extended Duration substrate (Thermo Fischer Scientific) and images were captured using Gel Doc system (Bio-Rad). Image Lab software (Bio-Rad) was used for densitometry of bands.

### Live-cell imaging and confocal microscopy

For cellular localization of lysosomes, cells were incubated with LysoTracker Red DND-99 (1:5,000) for 5 minutes at 37 °C followed by three times washing with warm HBSS. Plasma membrane and nuclei were stained by wheat germ agglutinin for 10 min and Höechst 33342 for 5 min, respectively, followed by three washes. The cells were suspended in warm OptiMEM and the live-cell imaging was performed by confocal microscopy at 40X objective using appropriate filter sets. The cytoplasmic distribution of lysosomes was determined by calculating the intensity of lysosome signal per unit area per cell in the field. The cell area was defined by plasma membrane staining.

Confocal microscopy was conducted following the procedure as previously described^23^. Cells were cultured in glass bottom 24-well plates. Cells were washed with PBS and fixed with 3.7% paraformaldehyde in PBS for 30 minutes at room temperature. After washing with PBS, samples were incubated with primary antibodies specific for LAMP1, LC3, TFEB, Calreticulin (CALR) or ORP5 diluted (1:100) in blocking buffer [1% BSA (w/v) and 0.1% TritonX-100 (v/v) in PBS] for overnight at 4 °C. After three washes with PBS, the cells were incubated with the corresponding fluorophore-conjugated Alexa secondary antibodies (1:100) diluted in blocking buffer at room temperature for 2 h and counterstained with 1 µM 4’,6-diamino-2-phenylindole dihydrochloride (DAPI; TCI). The samples were mounted in Prolong Gold Anti-fade reagent (P36930, Thermo Fisher Scientific), acquired on Nikon confocal laser scanning microscope (Center for Microscopy and Image Analysis, Meharry Medical College). The micrographs were processed with Adobe Photoshop software (version CS5, Adobe System, Inc., San Jose, CA). Quantitative image analysis was performed by drawing the ROI around the cell boundaries of 3–8 cells and measuring the signal intensity and area using the same parameters (i.e., pinhole, laser power, and offset gain and detector amplification below pixel saturation). The depth of LAMP1 staining in cells was measured using full confocal z-stacks (around 25) of each cell and the volume of LAMP1 staining was calculated by multiplying the area, depth and intensity for each cells. For co-localization studies, Nikon’s co-localization analytical tools were used to determine the Pearson coefficient of signals.

### Electron microscopy

All EM reagents were purchased from Electron Microscopy Sciences (Hatfield, PA). The cells were fixed in 2.5% glutaraldehyde in 0.1 M cacodylate for 1 hour. Samples were postfixed with 1% tannic acid in cacodylate for 1 hour and then 1% uranyl acetate in ddH_2_O for 30 minutes, followed by dehydration in a graded ethanol series. After dehydration the samples were transitioned to propylene oxide and infiltrated with a quetol 651 formulated Spurr’s resin^62^. The resin was polymerized at 60 °C for 48 hours. Thin sections were cut at a nominal thickness of 70 nm and poststained with 2% uranyl acetate and Reynold’s lead citrate. TEM imaging was performed using an FEI Technai T-12 transmission electron microscope operating at 100 kV using a side mounted AMT CCD camera.

### Subcellular distribution of unesterified cholesterol and neutral lipids

Cells cultured in glass bottom 24-well plates were fixed as previously described^23^. Cells were stained with Filipin III (12.5 μg/mL in PBS) for 45 min or Nile Red (100 ng/mL in PBS) for 15 min at room temperature. After washing three times with PBS, cells were mounted in anti-fade mounting medium. Images were acquired using a Nikon TE2000 wide field microscope with standard filter sets using 20X objective and analyzed using Nikon image software.

### Cholesterol content analysis

The levels of total and free cholesterol in cells were measured using fluorometric cholesterol assay kit as per manufacturer’s instructions (Cell Biolabs). Briefly, cells were lysed in chloroform/isopropanol/NP-40 (7:11:0.1) and debris was removed by centrifugation. The organic solvents were removed from samples by air drying at 50 °C for 1–2 h followed by vacuum drying for 2 h. The dried lipid content was dissolved in assay diluent. Super oxide dismutase (40 U/ml) was added to the samples to minimize the endogenous oxidation of assay probe. The cholesterol reaction mixture with or without cholesterol esterase was mixed with samples along with standards and incubated for 15–30 min at 37 °C. The amount of cholesterol was calculated by standard curve.

### Real-time PCR assay

Cells were incubated with CDs for 24 h before total RNA was extracted using QIAzol Lysis Reagent (Qiagen). The RNA samples were treated with RNase free DNase (Qiagen) and purified as per manufacturer’s instructions. The cDNA was synthesized from 1 μg of total RNA using iScript cDNA synthesis kit (Bio-Rad). Quantitative gene expression was performed in reaction containing cDNA, TaqMan Fast Advanced Master Mix, and the corresponding TaqMan gene expression probe with GAPDH as a reference probe in a CFX96 Real-Time PCR detection system (Bio-Rad) programmed for initial steps of 2 min at 50 °C and 2 min at 95 °C, and amplified for total 40 cycles of 10 s at 95 °C and 30 s at 60 °C. The threshold cycle (CT) was extracted from the PCR amplification plot using CFX manager software (Bio-Rad), and ΔCT values were calculated to evaluate the difference between the CT of a target gene and the CT of the housekeeping gene, GAPDH, as follows: ΔCT = CT (target gene) − CT (GAPDH). The relative mRNA expression levels of the CLEAR network in CD treated cells was normalized to those measured in untreated cells. Each data point was assayed in triplicate.

### Statistical analysis

Statistical analysis was conducted as previously described^23^. Results are expressed as mean ± standard error of mean (S.E.M.). For comparisons, the statistical significance of differences in mean values was determined by analysis of variance (ANOVA) using GraphPad Prism 7 (GraphPad software, La Jolla, CA). A *p*-value of 0.05 or less was considered statistically significant.

## Supplementary information

Supplementary information.
