# Supplementary material for: 2-Hydroxypropyl-gamma-cyclodextrin overcomes NPC1 deficiency by enhancing lysosome-ER association and autophagy
Source: Sci Rep. 2020 May 26;10:8663. doi: 10.1038/s41598-020-65627-4 (PMC7250861; doi:10.1038/s41598-020-65627-4)

Supplementary Information

2-Hydroxypropyl-gamma-cyclodextrin overcomes NPC1 deficiency by enhancing lysosome-ER association and autophagy

Ashutosh Singhal[1], Evan S. Krystofiak[2], W. Gray Jerome[3], and Byeongwoon Song[1*]

Figure S1. Effect of HPβCD and HPγCD on the morphology of healthy and NPC1 fibroblasts. Skin fibroblasts from a healthy donor (Healthy) or NPC1 patient (NPC1) were treated for 72 h with 1-40 mM of either HPγCD or HPβCD and subjected to microscopy. The images of the treated cells are shown. HPβCD exerted more potent cytotoxic activity in both healthy and NPC1 mutant cells compared to HPγCD. Data are a representative of three independent experiments.

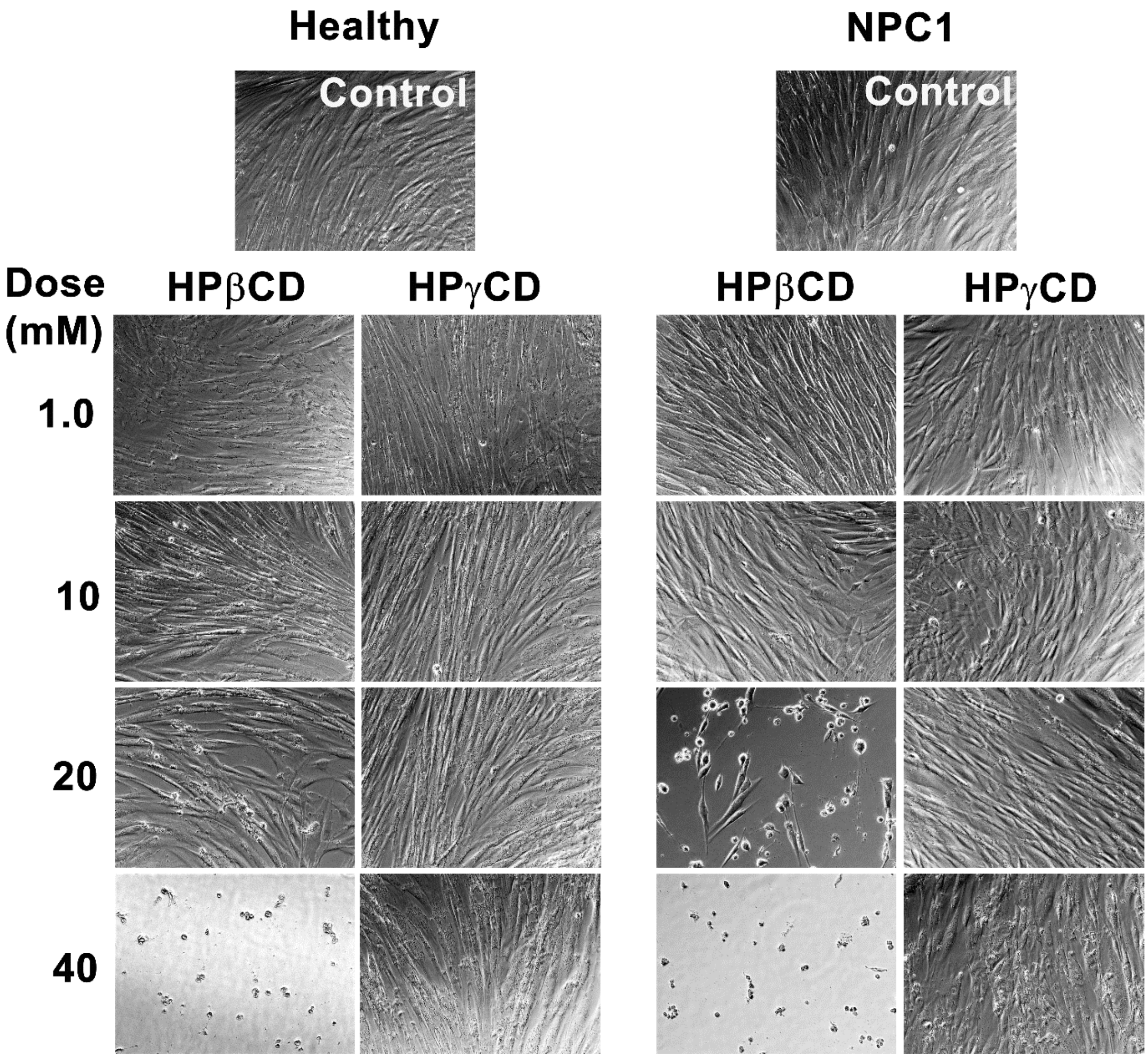

Figure S2. Effect of HPβCD and HPγCD on lysosome-mitochondria association in NPC1 fibroblasts. NPC1 mutant cells treated with HPγCD or HPβCD (1 mM, 72 h) were analyzed for lysosome-mitochondria association by confocal microscopy. The lysosomal marker LAMP1 (green) was partially co-localized with the mitochondria marker TOMM20 (red) as visualized by yellow color resulting from merged image of two proteins. HPγCD or HPβCD treatment did not increase the co-localization of lysosomes and mitochondria. Nuclei were stained using DAPI (blue). Data are a representative of three independent experiments. Scale bar = 50 μm.

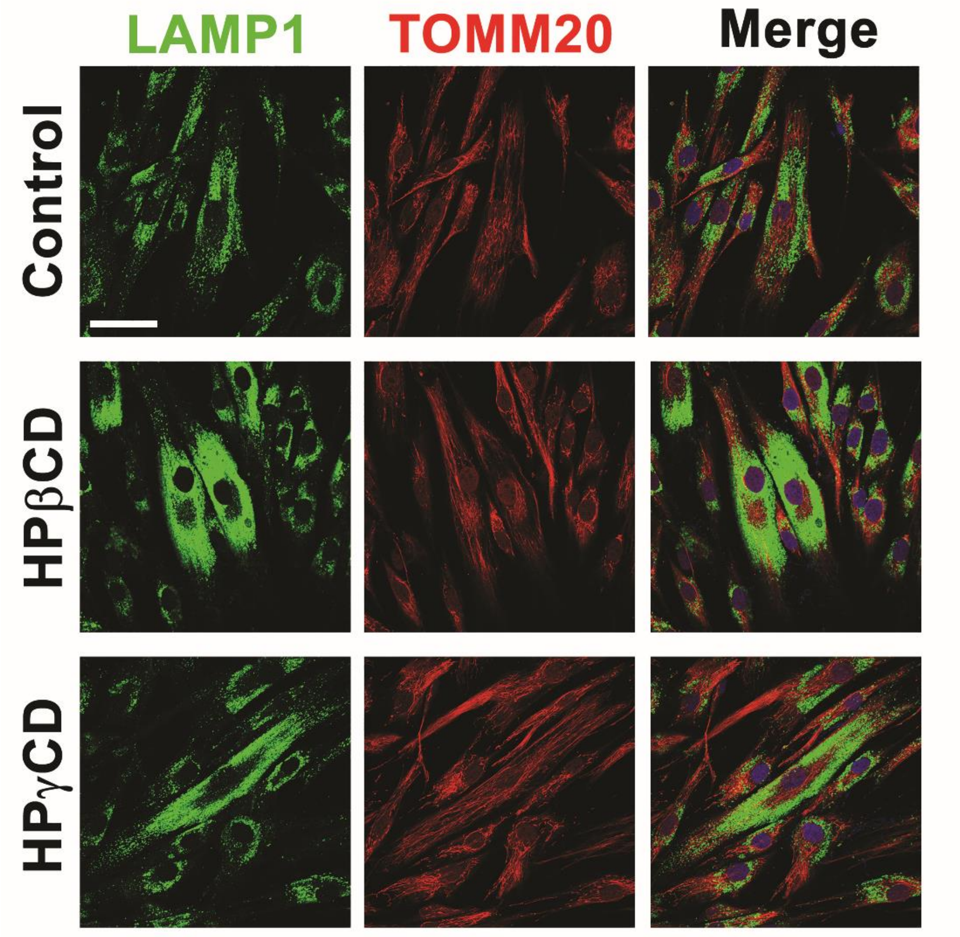

Figure S3. Effect of HPβCD and HPγCD on lysosome-peroxisome association in NPC1 fibroblasts. NPC1 mutant cells treated with HPγCD or HPβCD (1 mM, 72 h) were analyzed for lysosome-peroxisome association by confocal microscopy. The peroxisome membrane protein ABCD3 (red) was partially co-localized with the lysosomal membrane protein LAMP1 (green) as visualized by yellow color resulting from merged image of two proteins. HPγCD or HPβCD treatment did not increase the co-localization of lysosomes and peroxisomes. Nuclei were stained by DAPI (blue). Data are a representative of three independent experiments. Scale bar = 50 μm.

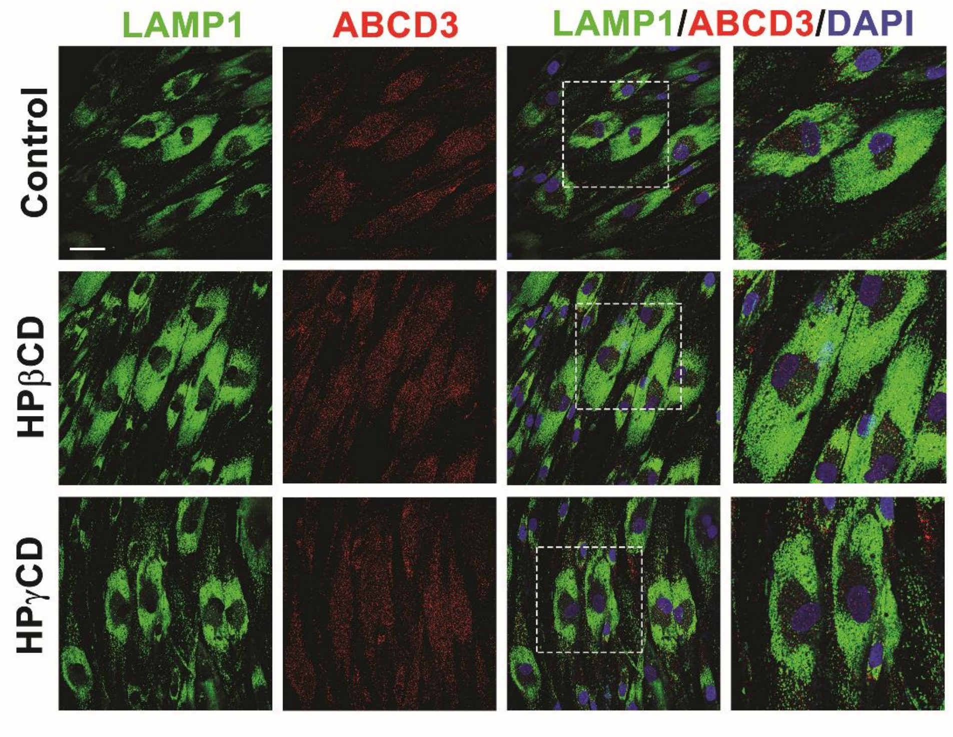

Figure S4. Effect of genistein on the viability of NPC1 fibroblasts. NPC1 mutant cells were treated with genistein (GNT) for 48 h at the indicated concentrations and subjected to cell viability analysis by using the CellTiter 96 Aqueous One Solution Cell Proliferation Assay System (Promega). The metabolic activity of untreated control cells was set as 100%. Data are mean ±SD of triplicates and a representative of three independent experiments. Symbols indicate the relative level of significance compared with control (***P<0.001).

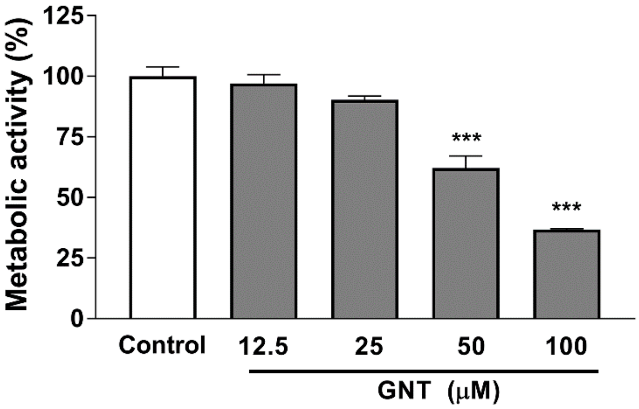

Supplement: Supplementary file 1 — Supplementary information. [file 41598_2020_65627_MOESM1_ESM.pdf]
